# A cohort study of low birth weight and health outcomes in the first year of life, Ghana

**DOI:** 10.2471/BLT.16.180273

**Published:** 2017-05-26

**Authors:** Maureen O’Leary, Karen Edmond, Sian Floyd, Sam Newton, Gyan Thomas, Sara L Thomas

**Affiliations:** a Department of Infectious Disease Epidemiology, Faculty of Epidemiology and Population Health, London School of Hygiene & Tropical Medicine, Keppel Street, London, WC1E 7HT, England.; b School of Paediatrics and Child Health, University of Western Australia, Crawley, Australia.; c Department of Community Health, Kwame Nkrumah University of Science and Technology, Kumasi, Ghana.; d Kintampo Health Research Centre, Kintampo, Ghana.

## Abstract

**Objective:**

To investigate the effect of birth weight on infant mortality, illness and care seeking in rural Ghana.

**Methods:**

Using randomized controlled trial data, we compared infants weighing 2.00–2.49, 1.50–1.99 and < 1.50 kg with non-low-birth-weight infants. We generated adjusted mortality hazard ratios (aHR), adjusted illness rate ratios (aRR) and adjusted odds ratios (aOR) for health-facility admissions and absence of care seeking for four time periods: infancy, the neonatal period, early infancy and late infancy – represented by ages of 0–364, 0–27, 28–182 and 183–364 days, respectively.

**Findings:**

Among 22 906 infants, compared with non-low-birth-weight infants: (i) infants weighing 2.00–2.49, 1.50–1.99 and < 1.50 kg were about two (aHR: 2.13; 95% confidence interval, CI: 1.76–2.59), eight (aHR: 8.21; 95% CI: 6.26–10.76) and 25 (aHR: 25.38; 95% CI: 18.36–35.10) times more likely to die in infancy, respectively; (ii) those born weighing < 1.50 kg were about 48 (aHR: 48.45; 95% CI: 32.81–71.55) and eight (aHR: 8.42; 95% CI: 3.09–22.92) times more likely to die in the neonatal period and late infancy, respectively; (iii) those born weighing 1.50–1.99 kg (aRR: 1.57; 95% CI: 1.27–1.95) or < 1.50 kg (aRR: 1.58; 95% CI: 1.13–2.21) had higher neonatal illness rates; and (iv) for those born weighing 1.50–1.99 kg, care was less likely to be sought in the neonatal period (aOR: 3.30; 95% CI: 1.98–5.48) and early infancy (aOR : 1.74; 95% CI: 1.26–2.39).

**Conclusion:**

For low-birth-weight infants in Ghana, strategies to minimize mortality and improve care seeking are needed.

## Résumé

**Objectif:**

Étudier l'effet du poids à la naissance sur la mortalité, la maladie et le recours à des soins de santé, pendant la période infantile, dans des régions rurales du Ghana.

**Méthodes:**

En utilisant les données d'un essai contrôlé randomisé, nous avons comparé les nourrissons nés avec un poids compris entre 2,00 et 2,49 kg, entre 1,50 et 1,99 kg et inférieur à 1,50 kg avec les nourrissons qui ne présentaient pas un faible poids à la naissance. Nous avons généré des ratios de risque de mortalité ajustés (RRa), des ratios de taux de maladies ajustés (RTa) et des rapports des cotes ajustés (RCa) pour les admissions dans des centres de santé et l'absence de recours à des soins, sur quatre périodes de vie: entre 0 et 364 jours, entre 0 et 27 jours, entre 28 et 182 jours et entre 183 et 364 jours.

**Résultats:**

Dans l’échantillon étudié de 22 906 nourrissons, comparativement à ceux qui ne présentaient pas un faible poids à la naissance: (i) les nourrissons nés avec un poids compris entre 2,00 et 2,49 kg, entre 1,50 et 1,99 kg et inférieur à 1,50 kg ont été associés, respectivement, à un risque deux fois supérieur (RRa: 2,13; intervalle de confiance (IC) de 95%: 1,76–2,59), huit fois supérieur (RRa: 8,21; IC 95%: 6,26–10,76) et 25 fois supérieur (RRa: 25,38; IC 95%: 18,36–35,10) de décès durant la première année de vie; (ii) ceux pesant moins d'1,50 kg à la naissance ont été associés à un risque environ 48 fois supérieur (RRa: 48,45; IC 95%: 32,81-71,55) et huit fois supérieur (RRa: 8,42; IC 95%: 3,09–22,92) de décès entre 0 et 27 jours et entre 183 et 364 jours respectivement; (iii) ceux nés avec un poids compris entre 1,50 et 1,99 kg (RTa: 1,57; IC 95%: 1,27–1,95) ou inférieur à 1,50 kg (RTa: 1,58; IC 95%: 1,13–2,21) ont été associés à des taux supérieurs de maladies durant les 27 premiers jours de vie; et (iv) pour les nourrissons nés avec un poids compris entre 1,50 et 1,99 kg, le recours à des soins de santé a été moins fréquent durant les 27 premiers jours de vie (RCa: 3,30; IC 95%: 1,98–5,48) et sur la période allant du 28^ème^ au 182^ème^ jour de vie (RCa: 1,74; IC 95%: 1,26-2,39).

**Conclusion:**

Au Ghana, pour les nourrissons nés avec un faible poids, des stratégies doivent être adoptées afin de réduire la mortalité et d’améliorer le recours aux soins.

## Resumen

**Objetivo:**

Investigar el efecto del peso al nacer en la mortalidad infantil, las enfermedades y la atención médica en la Ghana rural.

**Métodos:**

Utilizando datos de ensayos controlados aleatorios, se comparó a los recién nacidos con un peso de 2,00–2,49, 1,50–1,99 y < 1,50 kg con recién nacidos sin insuficiencia ponderal. Se generaron cocientes de riesgos instantáneos ajustados (aHR) relativos a la mortalidad, razones de tasa ajustadas (aRR) relativas a enfermedades y cocientes de posibilidades ajustados (CPa) para las admisiones en centros sanitarios y la ausencia de atención médica durante cuatro periodos de tiempo: infancia, período neonatal, primera infancia e infancia tardía, representados por las edades de 0–364, 0–27, 28–182 y 183–364 días, respectivamente.

**Resultados:**

Entre 22 906 recién nacidos, comparados con recién nacidos sin insuficiencia ponderal: (i) los recién nacidos con un peso de 2,00–2,49, 1,50–1,99 y < 1,50 kg tenían dos (aHR: 2,13; intervalo de confianza, IC, del 95%: 1,76–2,59), ocho (aHR: 8,21; IC del 95%: 6,26–10,76) y 25 (aHR: 25,38; IC del 95%: 18,36–35,10) veces más posibilidades de morir durante la infancia, respectivamente; (ii) aquellos que nacieron con un peso de < 1,50 kg tenían 48 (aHR: 48,45; IC del 95%: 32,81-71,55) y ocho (aHR: 8,42; IC del 95%: 3,09–22,92) veces más posibilidades de morir durante el período neonatal y la infancia tardía, respectivamente; (iii) los que nacieron con un peso de 1,50–1,99 kg (aRR: 1,57; IC del 95%: 1,27–1,95) o < 1,50 kg (aRR: 1,58; IC del 95%: 1,13–2,21) tenían las tasas de enfermedad neonatal más elevadas; y (iv) aquellos que nacieron con un peso de 1,50–1,99 kg, era menos probable que necesitaran atención médica en el período neonatal (CPa: 3,30; IC del 95%: 1,98–5,48) y en la primera infancia (CPa: 1,74; IC del 95%: 1,26–2,39).

**Conclusión:**

Se necesitan estrategias para minimizar la mortalidad y mejorar la atención médica de los recién nacidos con insuficiencia ponderal en Ghana.

## ملخص

**الغرض:**

التحقيق في تأثير وزن الولادة على وفيات الرضع والإصابة بالمرض وطلب الرعاية في المناطق الريفية بغانا.

**الطريقة:**

باستخدام بيانات التجارب العشوائية التي تمت مراقبتها، قمنا بمقارنة الرضع الذين يتراوح وزنهم ما بين 2.00 و2.49، و1.50 و1.99 والأقل من 1.50 كلغ مع الرضع غير منخفضي الوزن عند الولادة. وأصدرنا نسب وفيات معدلة (aHR)، ونسب معدلات المرض المعدلة (aRR)، ونسب الاحتمالية المعدلة (aOR) لحالات القبول في المرافق الصحية وغياب طلب الرعاية لمدة أربع فترات زمنية تتمثل في: الرضع، وفترة حديثي الولادة، ومرحلة الطفولة المبكرة، ومرحلة الطفولة المتأخرة - ممثلة بالأعمار 0-364، 0-27، 28-182، 183-364 يومًا، على التوالي.

**النتائج:**

من بين 22,906 أطفال تمت مقارنتهم بالأطفال غير منخفضي الوزن عند الولادة: (1) كان عدد الأطفال الذين يتراوح وزنهم ما بين 2.00–2.49، و1.50–1.99 والأقل من 1.50 كلغ حوالي اثنين (نسبة الوفيات المعدلة: 2.13؛ بنسبة أرجحية مقدارها ‏95‏‏%: 1.76-2.59)، وثمانية (نسبة الوفيات المعدلة: 8.21؛ ‏‏95% كمقدار لنسبة الأرجحية: 6.26-10.76) و25 (نسبة الوفيات المعدلة: 25.38؛ نسبة أرجحية تبلغ ‏95‏%: 18.36-35.10) مرة أكثر عرضة للموت في مرحلة الطفولة، على التوالي. (2) كان عدد أولئك الذين ولدوا ويقل وزنهم عن 1.50 كلغ حوالي 48 (نسبة الوفيات المعدلة: 48.45؛ ‏‏95% كمقدار لنسبة الأرجحية: 32.81 – 71.55) وثمانية (نسبة الوفيات المعدلة: 8.42؛ نسبة أرجحية تبلغ ‏95‏%: 3.09-22.92) مرة أكثر عرضة للموت في فترة حديثي الولادة والطفولة المتأخرة على التوالي؛ (3) كان عدد أولئك الذين يتراوح وزنهم ما بين 1.50 إلى 1.99 كلغ (معدل المرض المعدل: 1.57؛ ‏‏95% كمقدار لنسبة الأرجحية: 1.27-195) أو أقل من 1.50 كلغ (معدل المرض المعدل: 1.58؛ ‏‏95% كمقدار لنسبة الأرجحية: 1.13-2.21) كان لديهم معدلات مرض أعلى لحديثي الولادة؛ و(4) لأولئك الذين ولدوا وتتراوح أوزانهم ما بين 1.50 إلى 1.99 كلغ، كانت إمكانية الحصول على الرعاية أقل في فترة حديثي الولادة (نسبة الاحتمالية المعدلة: 3.30؛ ‏95% كمقدار لنسبة الأرجحية: 1.98-5.48) والطفولة المبكرة (نسبة الاحتمالية المعدلة: 1.74؛ نسبة أرجحية تبلغ ‏95‏%: 1.26–2.39).

**الاستنتاج:**

بالنسبة للرضع منخفضي الوزن في غانا، هناك حاجة إلى وضع استراتيجيات للحد من الوفيات وتحسين الرعاية الصحية.

## 摘要

**目的:**

旨在调查加纳乡村地区出生体重对婴儿死亡率、患病及获取护理的影响。

**方法:**

我们使用随机对照试验数据将出生体重为 2.00–2.49 kg、1.50–1.99 kg 以及 1.50 kg 以下婴儿与非出生体重偏低婴儿进行了对比。 我们生成了四个阶段（婴儿期、新生儿期、婴儿早期、及婴儿晚期 ——分别代表 0–364 天、0–27 天、28–182 天 、183–364 天年龄段的婴儿）中能够获取卫生设施护理的婴儿及缺乏护理的婴儿的调整后死亡率危险比率 (aHR) 、调整后患病率比率 (aRR) 及调整后比值比 (aOR)。

**结果:**

与非出生体重偏低婴儿相比，22 906 名婴儿中： (i) 出生体重为 2.00–2.49 kg、1.50–1.99 kg 及 1.50 kg 以下的婴儿，其婴儿期死亡的可能性分别高出两倍（aHR: 2.13；95% 置信区间，CI： 1.76–2.59），八倍 (aHR: 8.21; 95% CI: 6.26–10.76) 和 25 倍 (aHR: 25.38; 95% CI: 18.36–35.10)；(ii) 出生体重为  1.50 kg 以下的婴儿，其新生儿期及婴儿晚期死亡的可能性分别高出 48 倍 (aHR: 48.45; 95% CI: 32.81-71.55) 和八倍 (aHR: 8.42; 95% CI: 3.09–22.92)；(iii) 出生体重为 1.50–1.99 kg (aRR: 1.57; 95% CI: 1.27–1.95) 或出生体重为 1.50 kg (aRR: 1.58; 95% CI: 1.13–2.21) 以下的婴儿，其新生儿期的患病率更高；(iv) 对于出生体重为 1.50–1.99 kg 的婴儿来说，其在新生儿期 (aOR: 3.30; 95% CI: 1.98–5.48) 及婴儿早期获取护理的可能性更低 (aOR : 1.74; 95% CI: 1.26–2.39)。

**结论:**

对于加纳地区出生体重偏低的婴儿，仍需采取措施以降低其死亡率并改善其获取护理的状况。

## Резюме

**Цель:**

Изучить влияние веса при рождении на показатели младенческой смертности, заболеваемости и частоты обращения за медицинской помощью в сельских районах Ганы.

**Методы:**

Используя данные рандомизированных контролируемых испытаний, мы провели сравнение младенцев с весом 2,00–2,49, 1,50–1,99 и < 1,50 кг и младенцев с нормальным весом при рождении. Мы установили скорректированные коэффициенты риска смертности (сРС), скорректированные коэффициенты риска заболеваемости (сРЗ) и скорректированное отношение шансов (сОШ) для случаев госпитализации и отсутствия обращения за медицинской помощью в течение четырех периодов времени (младенчество, неонатальный период, ранний младенческий возраст и поздний младенческий возраст), представленных по возрастным категориям: 0–364, 0–27, 28–182 и 183–364 дня соответственно.

**Результаты:**

Среди 22 906 младенцев (при сравнении с младенцами, имевшими нормальный вес при рождении): (I) у младенцев, родившихся с весом 2,00–2,49, 1,50–1,99 и < 1,50 кг, вероятность младенческой смертности была выше приблизительно в два раза (сРС: 2,13; 95%-й доверительный интервал, ДИ: 1,76–2,59), в восемь раз (сРС: 8,21; 95%-й ДИ: 6,26–10,76) и в 25 раз (сРС: 25,38; 95%-й ДИ: 18,36–35,10); (II) у младенцев, родившихся с весом < 1,50 кг, вероятность смертности в неонатальный период и в период позднего младенческого возраста была выше примерно в 48 раз (сРС: 48,45; 95%-й ДИ: 32,81–71,55) и в восемь раз (сРС: 8,42; 95%-й ДИ: 3,09–22,92); (III) младенцы, родившиеся с весом 1,50–1,99 кг (сРЗ: 1,57; 95%-й ДИ: 1,27–1,95) или < 1,50 кг (сРЗ: 1,58; 95%-й ДИ: 1,13–2,21), имели более высокие показатели неонатальной заболеваемости; (IV) для младенцев, родившихся с весом 1,50–1,99 кг, частота обращения за медицинской помощью была ниже в неонатальный период (сОШ: 3,30; 95%-й ДИ: 1,98–5,48) и в период раннего младенческого возраста (сОШ: 1,74; 95%-й ДИ: 1,26–2,39).

**Вывод:**

В Гане для младенцев с низким весом при рождении необходима разработка стратегий для сведения к минимуму смертности и повышения мотивации для обращения за медицинской помощью.****

## Introduction

Approximately 14% of infants in low-income countries weigh less than 2.5 kg at birth – many are born preterm.^1^ Most research on mortality and illness among low-birth-weight infants has focused on the neonatal period^2^ and few studies from sub-Saharan Africa have generated population-based estimates of post-neonatal outcomes.^3^^–^^6^ Such estimates are particularly scarce for the – mostly preterm – infants born weighing less than 1.50 kg.^7^^,^^8^ Data from sub-Saharan Africa on the degree to which low birth weight increases the risk of mortality and illness in the post-neonatal period are lacking but are needed to target interventions. Caregivers who think an infant is likely to die may be less likely to seek care for the infant, especially if the infant is fragile and small and the affected household is poor and far from a health facility.^9^

We used data from a neonatal vitamin A supplementation (Neovita) trial to investigate birth weight as a risk factor for illness and mortality in infancy. Our primary objective was to determine the extent to which low-birth-weight infants were at increased risk of mortality and illness in the first year of life. Our secondary objectives were: (i) to assess, among sick infants, the association between birth weight and care seeking and health-facility admissions; (ii) to examine how the effects of birth weight on infant illness and mortality varied between the neonatal period, early infancy and late infancy: and (iii) to investigate whether any effect of birth weight on mortality varied by distance to the nearest health facility and/or socioeconomic status.

## Methods

The Neovita trial was conducted at the Kintampo Health Research Centre in rural Ghana.^10^^,^^11^ The study area is served by four district hospitals and 69 health facilities. All pregnancies and deliveries among women aged 15–49 years between August 2010 and November 2011 were identified through a population-based prospective surveillance system. Infants who were staying in the study area for at least six months after enrolment, who were aged less than four days and who were able to suck or feed at screening were enrolled.

Using calibrated electronic or spring scales, fieldworkers recorded birth weights to the nearest 0.1 and 0.2 kg, respectively. Over 70% of enrolled infants were weighed within 24 hours of delivery and only five (0.2%) were weighed more than 72 hours after delivery.

During both pregnancy surveillance and at enrolment, fieldworkers asked each pregnant woman the date of her last menstrual period. Various household, infant and maternal characteristics were recorded at enrolment.^10^^,^^11^ Infants were visited monthly for the first year of life. At each visit, the infant’s mother was asked if the infant had been ill since the previous visit and, if so, when the illness had started and ended, whether care for the illness had been sought and, if so, whether the infant had been admitted, for at least one night, to a health facility. Data were also collected on the infant’s vital status and, when applicable, date of death.

The primary outcomes were illness and mortality in the first year of life. For each reported episode of illness, we investigated absence of care seeking and admission to a health facility.

If an end date but no start date was recorded for an illness, the start date was assumed to have been five days, i.e. the median duration of illness recorded, before the end date. If both start and end dates were unrecorded, the start date was assumed to be the midpoint between the date the illness was reported and the date of the previous follow-up visit. Similarly, if a study infant died within a year of birth but its date of death was not recorded, it was assumed to have died at the midpoint between the first report of its death and the last report of it being alive. Facility admissions occurring within 28 days of a previous admission were reviewed to assess whether they represented responses to a single ongoing illness.

The primary exposure, birth weight, was divided into non-low-birth-weight, i.e. at least 2.50 kg, and three categories of low-birth-weight: 2.00–2.49, 1.50–1.99 and less than 1.50 kg.^7^^,^^8^^,^^12^

For most of the 28 498 pregnancies identified during the study, data on last menstrual period were missing (*n* = 16 398) or inconsistently reported (*n* = 1935). Given this and the known discordance between mothers’ reports of the dates of their last menstrual periods and the corresponding gestational ages assessed by ultrasonography,^13^^–^^15^ we did not investigate the association between gestational age and our outcomes of interest.

For all analyses, follow-up started at birth and ended at: (i) 364 days of age; (ii) the date of death, for infants who died when aged less than 365 days; or (iii) the last date the infant was seen alive if they exited the study when aged less than 365 days.

### Data analysis

All analyses were conducted using Stata version 13.1 (StataCorp. LP, College Station, United States of America).

We generated Kaplan–Meier curves of the probability of survival for low-birth-weight infants compared with non-low-birth-weight infants. We calculated mortality rates for the first year of life. As mortality rate changes rapidly, particularly in the neonatal period, we used multivariable Cox regression to calculate adjusted hazard ratios (aHR) for the association between birth weight and mortality.

To allow for repeated illness events, we used random-effects Poisson regression to calculate adjusted rate ratios (aRR) for the association between birth weight and infant illness.

Among infants with reported illness, for each illness episode, we used random-effects logistic regression to calculate adjusted odds ratios (aOR) of the association between birth weight and an absence of care seeking or health-facility admission. For each analysis, we assessed whether the effect of birth weight varied between the neonatal, early and late infant periods – represented by ages of 0–27, 28–182 and 183–364 days, respectively – by fitting birth weight as an interaction term with time period. Similarly, for mortality, we assessed whether the effect of birth weight varied by distance to the nearest health facility and/or socioeconomic status.

For all analyses, we used likelihood ratio tests and 95% confidence intervals (CI) to assess the statistical evidence for an association between birth weight and each outcome. We also adjusted a priori for infant sex and single/multiple birth, maternal age, education, illness and occupation and household exposure to indoor smoke, distance to nearest health facility, ethnicity, number of children in family, religion and socioeconomic status.

### Ethics

Ethics approval for the collection of data included in this study was granted by the Ethics Committees of the World Health Organization, the London School of Hygiene & Tropical Medicine and the Kintampo Health Research Centre.

## Results

Of the 22 955 infants enrolled in the trial, we included the 22 906 (99.8%) with complete covariate data in our analyses. Of these, almost 16% (3584) were low-birth-weight (Table 1).

**Table 1:** Characteristics of 22 906 infants included in the analyses, Ghana, 2010–2011

Characteristic	No. (%)
**Birth weight in kg**	
≥ 2.50	19 322 (84.4)
2.00–2.49	3 023 (13.2)
1.50–1.99	444 (1.9)
< 1.50	117 (0.5))
**Religion of head of household**	
Christian	15 961 (69.7)
Muslim	5 486 (24.0)
Other	1 459 (6.4)
**Ethnicity of household**	
Akan	10 690 (46.7)
Other	12 216 (53.3)
**Maternal education**	
None	7 101 (31.0)
Primary	4 232 (18.5)
Post-primary	11 573 (50.5)
**Maternal occupation**	
Farming	6 642 (29.0)
Government, private or other employed	1 223 (5.3)
Self-employed	8 934 (39.0)
Not employed	6 107 (26.7)
**Socioeconomic status, as wealth quintile**	
1 (poorest)	4 489 (19.6)
2	4 539 (19.8)
3	4 576 (20.0)
4	4 638 (20.2)
5 (richest)	4 664 (20.4)
**Exposure to indoor smoke**	
Exposed	13 033 (56.9)
**Place of delivery**	
Facility	17 552 (76.6)
Non-facility	5 354 (23.4)
**Distance to nearest health facility in km**	
< 1.00	13 856 (60.5)
1.00–4.99	5 282 (23.1)
≥ 5.00	3 768 (16.4)
**Maternal age in years**	
15–19	2 644 (11.5)
20–24	5 880 (25.7)
25–29	6 149 (26.8)
30–34	4 611 (20.1)
≥ 35	3 622 (15.8)
**No. of children in family**	
0–1	6 722 (29.3)
2–3	9 137 (39.9)
≥ 4	7 047 (30.8)
Unknown	
**Maternal illness**	
Present	1 122 (4.9)
**Infant sex**	
Female	11 286 (49.3)
**Single or multiple birth**	
Multiple	845 (3.7)

### Mortality

Of the included infants, 698 (3.0%) died younger than 365 days. Of these 698 deaths, 277 (39.7%) occurred in the neonatal period, 248 (35.5%) in early infancy and 173 (24.8%) in late infancy. The numbers of deaths per 1000 live births were 30.5 overall and 22.4, 48.6, 160.0 and 402.0 among infants born weighing at least 2.50, 2.00–2.49, 1.50–1.99 and less than 1.50 kg, respectively

**Fig. 1:**
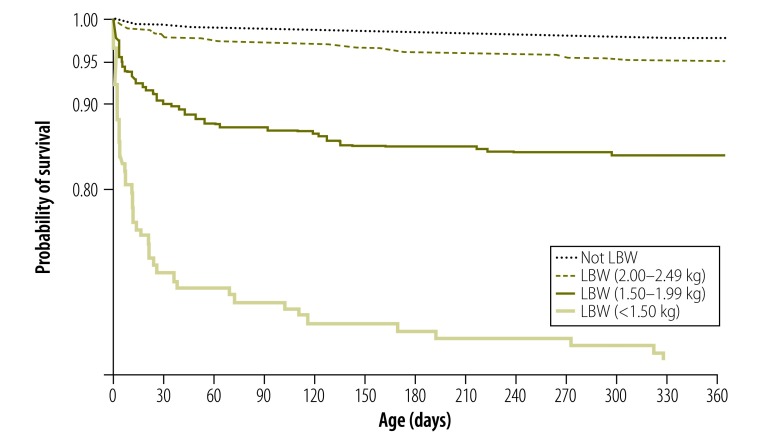
Probability of survival in the first year of life, by birth weight, Ghana, 2010–2011

Mortality declined with age but was consistently higher for low-birth-weight infants than for non-low-birth-weight infants (Fig. 1). The likelihood of death increased with lower birth weight (*P* < 0.0001). After adjusting for all potential confounders, infants born weighing 2.00–2.49, 1.50–1.99 and less than 1.50 kg were about two (aHR: 2.13), eight (aHR: 8.21), and 25 (aHR: 25.38) times more likely to die in their first year of life than non-low-birth-weight infants (Table 2).

LBW: low birth weight.

**Table 2:** Associations between birth weight and infant mortality, illness, absence of care seeking and health-facility admission in the first year of life Ghana, 2010–2011

Variable	Value for infants with birth weight of:			
	≥ 2.5 kg	2.00–2.49 kg	1.50–1.99 kg	< 1.50 kg
**Mortality**				
No. of deaths (no. of PDOFU)	433 (7 326 996)	147 (1 119 524)	71 (146 813)	47 (29 181)
Deaths/1000 YOFU	21.6 (19.6–23.7)	48.0 (40.8–56.4)	176.6 (140.0–222.9)	588.3 (442.0–783.0)
Hazard ratio (95% CI)				
Crude	Ref	2.21 (1.83–2.66)	7.92 (6.16–10.18)	24.51 (18.13–33.12)
Adjusted^a^	Ref	2.13 (1.76–2.59)	8.21 (6.26–10.76)	25.38 (18.36–35.10)
**Illness**				
No. of episodes (no. of PDOFU)	47 969 (6 832 406)	7 379 (1 041 876)	1 029 (136 089)	233 (26 638)
Episodes/1000 YOFU	2 564.4 (2 541.5–2 587.4)	2 586.9 (2 528.5–2 646.6)	2 761.7 (2 598.1–2 935.7)	3 194.9 (2 809.9–3 632.6)
Relative risk (95% CI)				
Crude	Ref	1.01 (0.98–1.04)	1.10 (1.01–1.19)	1.32 (1.12–1.57)
Adjusted^a^	Ref	0.99 (0.96–1.03)	1.06 (0.98–1.14)	1.15 (0.98–1.36)
**Absence of care seeking**				
No. of absences (no. of illness episodes)	7 680 (48 115)	1 214 (7 405)	235 (1 031)	52 (236)
Percentage of illness episodes without care seeking (95% CI)	16.0 (15.6–16.3)	16.4 (15.6–17.3)	22.8 (20.3–25.5)	22.0 (17.2–27.8)
Odds ratio (95% CI)				
Crude	Ref	1.03 (0.95–1.12)	1.72 (1.41–2.08)	1.76 (1.18–2.63)
Adjusted^a^	Ref	1.00 (0.91–1.09)	1.46 (1.18–1.81)	1.05 (0.68–1.63)
**Health-facility admission**				
No. of admissions (no. of illness episodes)	3 496 (48 115)	580 (7 405)	88 (1 031)	23 (236)
Percentage of illness episodes with admission (95% CI)	7.3 (7.0–7.5)	7.8 (7.2–8.5)	8.5 (7.0–10.4)	9.7 (6.6–14.2)
Odds ratio (95% CI)				
Crude	Ref	1.10 (0.98–1.23)	1.16 (0.88–1.52)	1.46 (0.86–2.48)
Adjusted^a^	Ref	1.12 (1.00–1.26)	1.12 (0.84–1.48)	1.41 (0.82–2.43)

CI: confidence interval; PDOFU: person-days of follow-up; Ref: reference; YOFU: years of follow-up.

^a^ Adjusted a priori for infant sex and single/multiple birth, maternal age, education, illness and occupation and household exposure to indoor smoke, distance to nearest health facility, ethnicity, number of children, religion and socioeconomic status.

We observed strong evidence that the effect of birth weight varied with time period (*P* < 0.0001; Table 3). Although higher mortality with lower birth weight was seen in each time period, the magnitude of the association declined over time (Table 3). For example, compared with non-low-birth-weight infants, infants born weighing less than 1.50 kg had about 48 times the mortality rate in the neonatal period (aHR: 48.45) but only eight times in late infancy (aHR: 8.42). The corresponding ratios were similar for infants born weighing 1.50–1.99 kg – 14.71 in the neonatal period and 1.61 in the late infant period – and, to a lesser extent, for the infants born weighing 2.00–2.49 kg – 2.29 and 1.60, respectively.

**Table 3:** Associations between birth weight and infant mortality, illness, absence of care seeking and health-facility admission in the neonatal period and early and late infancy, 2010–2011

Variable, time period**^a^**	Value for infants with birth weight of:			
	≥ 2.5 kg	2.00–2.49 kg	1.50–1.99 kg	< 1.50 kg
**Mortality, neonatal period**				
No. of deaths (no. of PDOFU)	144 (51 8 319)	53 (80 502)	45 (11 146)	35 (2 502)
Deaths/1000 YOFU	97.9 (83.1–115.2)	231.9 (177.2–303.6)	1357.1 (1 006.5–1 829.9)	4947.3 (3 552.1–6 890.4)
Adjusted hazard ratio (95% CI)^b^	Ref	2.29 (1.66–3.15)	14.71 (10.37–20.86)	48.45 (32.81–71.55)
**Mortality, early infancy**				
No. of deaths (no. of PDOFU)	157 (2 884 213)	61 (442 344)	22 (57 927)	8 (11 717)
Deaths/1000 YOFU	19.1 (16.3–22.4)	47.1 (36.3–61.0)	151.2 (101.4–225.6)	249.6 (124.8–499.1)
Adjusted hazard ratio (95% CI)^b^	Ref	2.45 (1.81–3.31)	7.22 (4.57–11.42)	12.95 (6.30–26.60)
**Mortality, late infancy**				
No. of deaths (no. of PDOFU)	132 (3 450 857)	33 (522 731)	4 (67 449)	4 (12 897)
Deaths/1000 YOFU	14.1 (11.8–16.7)	23.2 (16.5–32.6)	21.8 (8.2–58.0)	113.9 (42.8–303.6)
Adjusted hazard ratio (95% CI)^b^	Ref	1.60 (1.09–2.35)	1.61 (0.59–4.39)	8.42 (3.09–22.92)
**Illness, neonatal period**				
No. of episodes (no. of PDOFU)	2 343 (537 087)	411 (83 316)	106 (11 533)	42 (2 534)
Episodes/1000 YOFU	1 593.4 (1 530.2–1 659.2)	1 801.8 (1 635.8–1 984.7)	3 357.0 (2 775.1–4 061.0)	6 053.9 (4 473.9–8 191.7)
Adjusted relative risk (95% CI)^b^	Ref	1.00 (0.89–1.12)	1.57 (1.27–1.95)	1.58 (1.13–2.21)
**Illness, early infancy**				
No. of episodes (no. of PDOFU)	14 644 (2 882 964)	2246 (441 850)	324 (57 927)	67 (11 543)
Episodes/1000 YOFU	1 855.3 (1 825.5–1 885.6)	1 856.6 (1 781.4–1 935.0)	2 042.9 (1 832.2–2 277.9)	2 120.1 (1 668.7–2 693.7)
Adjusted relative risk (95% CI)^b^	Ref	0.99 (0.94–1.04)	1.10 (0.97–1.23)	1.10 (0.85–1.43)
**Illness, late infancy**				
No. of episodes (no. of PDOFU)	30 776 (341 2047)	4 689 (516 711)	596 (66 629)	122 (12 561)
Episodes/1000 YOFU (95% CI)	3 294.5 (3 257.9–3331.5)	3 314.5 (3 221.0–3 410.8)	3 267.2 (3 015.1–3 540.3)	3 547.5 (2 970.7–4 236.3)
Adjusted relative risk (95% CI)^b^	Ref	0.99 (0.96–1.03)	0.99 (0.89–1.08)	1.07 (0.87–1.32)
**Absence of care seeking, neonatal period**				
No. of absences (no. of illness episodes)	1 210 (2 378)	217 (420)	78 (107)	30 (44)
Percentage of illness episodes without care seeking (95% CI)	50.9 (48.9–52.9)	51.7 (46.9–56.4)	72.9 (63.7–80.5)	68.2 (53.0–80.3)
Adjusted odds ratio (95% CI)^b^	Ref	1.04 (0.81–1.34)	3.30 (1.98–5.48)	2.07 (0.97–4.43)
**Absence of care seeking, early infancy**				
No. of absences (no. of illness episodes)	2 549 (15 227)	403 (2 331)	82 (333)	8 (70)
Percentage of illness episodes without care seeking (95% CI)	16.7 (16.2–17.3)	17.3 (15.8–18.9)	24.6 (20.3–29.5)	11.4 (5.8–21.3)
Adjusted odds ratio (95% CI)^b^	Ref	1.03 (0.90–1.19)	1.74 (1.26–2.39)	0.63 (0.27–1.46)
**Absence of care seeking, late infancy**				
No. of absences (no. of illness episodes)	3 921 (30 510)	594 (4 654)	75 (591)	14 (122)
Percentage of illness episodes without care seeking (95% CI)	12.9 (12.5–13.2)	12.8 (11.8–13.8)	12.7 (10.2–15.6)	11.5 (6.9–18.5)
Adjusted odds ratio (95% CI)^b^	Ref	0.97 (0.86–1.09)	0.98 (0.72–1.32)	0.85 (0.43–1.66)
**Health-facility admission, neonatal period**				
No. of admissions (no. of illness episodes)	250 (2 378)	48 (420)	7 (107)	6 (44)
Percentage of illness episodes with admission (95% CI)	10.5 (9.3–11.8)	11.4 (8.7–14.8)	6.5 (3.1–13.1)	13.6 (6.2–27.4)
Adjusted odds ratio (95% CI)^b^	Ref	1.11 (0.77–1.60)	0.56 (0.24–1.29)	1.59 (0.59–4.27)
**Health-facility admission, early infancy**				
No. of admissions (no. of illness episodes)	1 019 (15 227)	194 (2 331)	28 (333)	6 (70)
Percentage of illness episodes with admission (95% CI)	6.7 (6.3–7.1)	8.3 (7.3–9.5)	8.4 (5.9–11.9)	8.6 (3.9–17.9)
Adjusted odds ratio (95% CI)^b^	Ref	1.35 (1.12–1.63)	1.19 (0.75–1.89)	1.34 (0.51–3.52)
**Health-facility admission, late infancy**				
No. of admissions (no. of illness episodes)	2 227 (30 510)	338 (4 654)	53 (591)	11 (122)
Percentage of illness episodes with admission (95% CI)	7.3 (7.0–7.6)	7.3 (6.6–8.0)	9.0 (6.9–11.6)	9.0 (5.1–15.6)
Adjusted odds ratio (95% CI)^b^	Ref	1.03 (0.89–1.18)	1.24 (0.88–1.75)	1.36 (0.65–2.85)

CI: confidence interval; PDOFU: person-days of follow-up; Ref: reference; YOFU: years of follow-up.

^a^ The neonatal period, early infancy and late infancy were represented by ages of 0–364, 0–27, 28–182 and 183–364 days, respectively.

^b^ Adjusted a priori for infant sex and single/multiple birth, maternal age, education, illness and occupation and household exposure to indoor smoke, distance to nearest health facility, ethnicity, number of children, religion and socioeconomic status.

The effect of birth weight on mortality did not vary by either distance to the nearest health facility or socioeconomic status – with *P*-values above 0.2 for all the relevant interactions.

### Infant illness

**Fig. 2:**
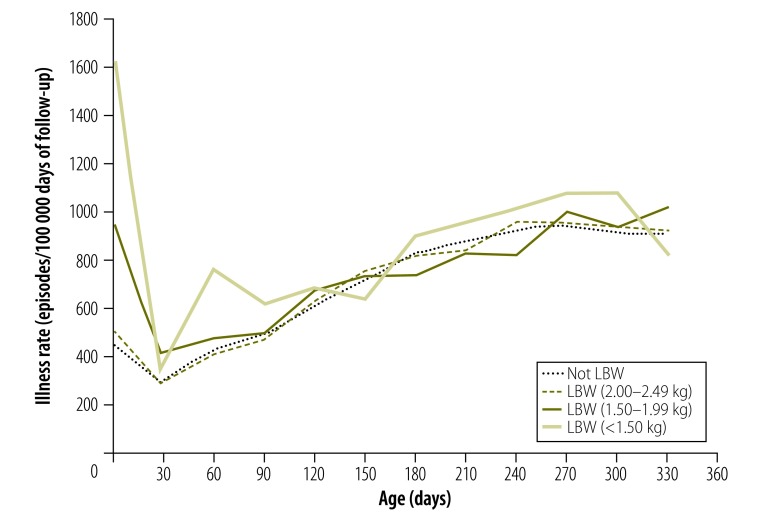
Illness rates in the first year of life, by birth weight, Ghana, 2010–2011

Mothers reported 56 610 episodes of illness in 19 292 infants. Following an initial peak in the neonatal period, age-specific illness rates increased over time (Fig. 2). Upon adjustment for other factors, birth weight was not associated with infant illness overall (Table 3) although the association varied significantly with time period (*P* = 0.0013). Compared with non-low-birth-weight infants, infants born weighing 1.50–1.99 kg (aRR: 1.57) and less than 1.50 kg (aRR: 1.58) had higher illness rates in the neonatal period – although there was little evidence of an association later in infancy (Table 3).

LBW: low birth weight.

### Care seeking

We observed evidence of an absence of care seeking for infants born weighing 1.50–1.99 kg (aOR, 1.46) – compared with ill non-low-birth-weight infants (Table 2). Although, in the univariable analysis, there was also evidence of an absence of care seeking for infants born weighing less than 1.50 kg (crude odds ratio, cOR: 1.76), this association was no longer apparent after adjustment for infant age and other covariates (aOR: 1.05; Table 2). Care seeking varied between the neonatal period and early and late infancy (*P* = 0.0002). However, in each of these time periods, an absence of care seeking was only observed for infants born weighing 1.50–1.99 kg.

### Admissions

Overall, 4187 admissions were reported for 3485 infants. We found no association between birth weight and admissions over the first year of life (Table 2) or in the neonatal, early or late infant periods (*P* = 0.1383).

### Additional analyses

To understand further how illness, care seeking and admissions related to mortality, we undertook additional post-hoc exploratory analyses of verbal postmortem data for the infants who died. We compared disease progression, care seeking and admissions during the fatal illness for the low-birth-weight and non-low-birth-weight infants, using proportions and *χ^2^* tests (Table 4). Data on cause of death were unavailable.

**Table 4:** Illness, care seeking and caregiving behaviour during fatal illnesses among infants in their first year of life, Ghana, 2010–2011

Variable	No. (%)		*P*
	Non-LBW infants (*n* = 422)	LBW infants (*n* = 262)	
**Duration of fatal illness in days**			
< 1	21 (5.0)	12 (4.6)	
1–7	216 (51.2)	142 (54.2)	
> 7	185 (43.8)	108 (41.2)	0.7440
**Care sought for infant?**			
No	55 (13.0)	60 (22.9)	
Yes	367 (87.0)	202 (77.1)	0.0010
**Days ill before care sought**^a^			
< 1	135 (36.8)	71 (35.2)	
1–3	174 (47.4)	86 (42.6)	
> 3	58 (15.8)	45 (22.3)	0.1510
**PMC sought**^a^			
No	24 (6.5)	29 (14.4)	
Yes	343 (93.5)	173 (85.6)	0.0020
**Sought care elsewhere**^a^			
No	228 (62.1)	116 (57.4)	
Yes	139 (37.9)	86 (42.6)	0.2730
**Caregiving for infants for whom PMC was sought**^b^			
Admitted to a health facility?			
No	179 (52.2)	82 (47.4)	
Yes	164 (47.8)	91 (52.6)	0.3040
Medical therapy received?			
No	52 (15.2)	38 (22.0)	
Yes^c^	291 (84.8)	135 (78.0)	0.0540
Died in health facility?			
No	162 (47.2)	88 (50.9)	
Yes	181 (52.8)	85 (49.1)	0.4350

LBW: low-birth-weight; PMC: professional medical care.

^a^ The denominators for the percentages shown were the numbers of infants for whom care was sought: 367 for the non-LBW and 202 for the LBW.

^b^ The denominators for the percentages shown were the numbers of infants for whom professional medical care was sought: 343 for the non-LBW and 173 for the LBW.

^c^ Medicine was prescribed and/or surgery occurred.

Verbal postmortem data were available for 684 (98.0%) of the 698 infants who died. Families of the low-birth-weight infants who died were less likely to have sought care than those of non-low-birth-weight infants who died (*P* = 0.001; Table 4). Among the families who did seek care, only 173 (85.6%) of the 202 families of low-birth-weight infants – compared with 343 (93.5%) of the 367 families of non-low-birth-weight infants (*P* = 0.002) – sought professional medical care – i.e. from a clinic, doctor, hospital, nurse or pharmacy. There was little evidence of differences – between the low-birth-weight and other infants who died – in the duration of illness, in the time to seek care, in the proportions of families who sought care from non-medically trained sources and in the proportions of infants who were admitted to a health facility or who died in a health facility (Table 4).

## Discussion

Low birth weight affects adversely on health outcomes throughout infancy. Compared with other infants, low-birth-weight infants – especially those born weighing less than 1.50 kg – have substantively higher mortality rates. In our study population, this association did not vary by socioeconomic status or by distance to the nearest health facility. Furthermore, low-birth-weight infants had higher illness rates in the neonatal period but care was less likely to be sought for them when they were ill in the neonatal period or early infancy – even if they were having illnesses that led to their deaths.

Although several studies have investigated the association between mortality and low birth weight in sub-Saharan Africa,^5^^,^^16^^–^^21^ few have generated population-based mortality estimates for infants born weighing either 1.50–1.99 kg or less than 1.50 kg. In a single study from Malawi from more than 20 years ago, neonatal and infant mortality rates were 13 and five times higher, respectively, among those with birth weights below 2.00 kg than in those with higher birth weights.^21^ These ratios are similar to our estimates for infants born weighing 1.50–1.99 kg. A birth weight of less than 1.50 kg may be considered a sensitive and specific marker for preterm birth.^22^^,^^23^ We compared our results for infants with such very low birth weights with those of two studies^6^^,^^24^ that investigated mortality among infants that were preterm and small for gestational age. In low- or middle-income countries, compared with other infants, infants who were both preterm and small for gestational age were found to be over 16, 19 and six times more likely to die during the early neonatal, late neonatal and post-neonatal periods, respectively.^6^ In the United Republic of Tanzania, compared with other infants, infants who were both preterm and small for gestational age were found to be 15 and three times more likely to die in the neonatal and post-neonatal periods, respectively.^24^ The effect estimates produced in both of these earlier studies are substantially lower than our related estimates – of 48-, 13- and eightfold higher risks of mortality, for infants born weighing less than 1.50 kg than for non-low-birth-weight infants, in the neonatal period, early infancy and late infancy, respectively

The association observed between birth weight and mortality in our study was not reflected in corresponding associations with illness – except in the neonatal period – or facility admissions. Our data indicate relatively low frequencies of care seeking for ill low-birth-weight infants, even for those suffering fatal illnesses. Lack of care seeking for such infants decreases their opportunity for hospital admission.

A few studies have investigated the association between birth weight and illness in Africa, with varying results. Several studies have reported no association between birth weight and infant clinic attendance, admissions or illness.^3^^,^^25^^–^^28^ In contrast, an analysis of hospital admissions – based on both written records and maternal recall – in a periurban area in the United Republic of Tanzania^29^ found that infants with birth weights of 2.00 kg or less were more likely to be hospitalized in the first year of life than infants with higher birth weights (aHR; 2.74; 95% CI: 1.66–4.54). As this analysis was restricted to admissions to district hospitals, the severity of the illnesses among those admitted was likely to be greater than in our analysis, which included admissions to any type of health facility. In urban South Africa, infants born at less than 32 weeks’ gestation were more likely to be hospitalized for respiratory syncytial virus, bronchiolitis and pneumonia in childhood than other infants.^30^ In another South African study, pneumonia was associated with preterm delivery – but not with low birth weight.^3^

We found that the families of sick low-birth-weight infants were less likely to have sought care for their infants, even when those infants were suffering from illnesses that led to their deaths. We are aware of only one study that has investigated this topic: an analysis of 840 infants, in rural Malawi, in which preterm and term infants were found to have accessed health care a similar number of times when investigated at 12, 18 and 24 months of age (*P* = 0.86).^25^

Several factors may explain why, in a population where birth weight was strongly associated with death, there was little association between birth weight and either care seeking or admissions for illness. First, our data on illness and any associated care seeking and admissions were based on maternal recall. As caregiver recognition of childhood illnesses in low- and middle-income settings is often poor,^31^ illness may have been underreported in our study. The possibility that illness in low-birth-weight infants was less, or more likely to be reported by mothers than illness in non-low-birth-weight infants cannot be excluded. Qualitative data from Uganda indicated poor recognition of low birth weight as a danger sign and a consequent lack of care seeking for neonatal illness.^32^ A failure to recognize and appreciate the severity of illness among low-birth-weight infants who subsequently die has also been reported.^33^

Compared with general illness, health-facility admission is probably a more notable event that is less likely to be underreported and is a useful marker for severe disease. Although severity is recognized as an important determinant of care seeking,^31^ in our study area it has been observed that care is not sought for up to 50% of severe illnesses.^34^ The apparent reluctance of caregivers in this area to seek care for sick low-birth-weight infants may explain the discordance between our reported admission and mortality rates. Caregivers may think that care seeking for weak low-birth-weight infants is pointless because they believe that health care will not increase the infant’s chance of surviving.^9^ In our study area certain illnesses are considered to be untreatable by modern medicine.^34^ The possibility that, in our study areas, such illnesses occur more frequently among low-birth-weight infants than other infants cannot be excluded.

Another possibility is that, during fatal illnesses, sudden illness onset and rapid disease progression are relatively common among low-birth-weight infants – leaving insufficient time to seek care before death. Although our analyses of fatal illnesses indicated that birth weight had no impact on illness duration or the time taken to seek care, the power of these analyses was limited by the small sample size.

This study has several strengths. Given the population-based nature of the cohort and the low numbers of individuals excluded from the analyses, our results are likely to be largely representative of the study area’s population. The large sample size provided sufficient power for us to generate estimates of mortality for several categories of low birth weight, including birth weights of less than 1.50 kg. Our study further benefited from low rates of loss to follow-up and from the almost complete data on mortality, including date of death. Any misclassification of deaths by time period should have been negligible.

This study has some limitations. As we lacked accurate data on gestational age at birth, we could not generate separate estimates by level of prematurity or for infants that were small for gestational age. We had fairly robust – albeit recall-based – data on whether care was sought for an infant during illness. However, data on several factors that could differ according to birth weight – e.g. time to illness onset, time to care seeking, disease severity and the type of care sought – other than admission to a health facility – were not collected. These factors merit further study. Despite the large sample size, the study was not sufficiently powered to detect moderate differences in illness or admissions by birth weight, especially in the smaller categories of birth weight stratified by time period. The recall of dates of illness was generally poor – e.g. the start dates of more than 40% of reported episodes of illness were recorded as unknown. Although the assumptions we made in estimating the missing dates may have led to some misclassification by time period, the monthly data collection meant that almost 75% of all imputed illness dates were reported within 30 days of a previous visit.

In conclusion, strategies to minimize neonatal and infant mortality should target the entire first year of life of low-birth-weight infants. Care for such infants needs to be improved. Our study highlights the need for further studies in Africa to investigate the association between birth weight and infant illness and mortality and any related caregiving and care seeking. Qualitative research on the care of low-birth-weight infants, including the barriers to – and facilitators of – care seeking, is needed.
